# Seroprevalence and risk factors of Toxoplasmosis among HIV infected women of child-bearing age attending care and treatment clinics in Dar es Salaam, Tanzania

**DOI:** 10.4314/ahs.v22i4.53

**Published:** 2022-12

**Authors:** Upendo O Kibwana, Joel Manyahi, Lilian B Nkinda, Dominic S Renatus, Doreen D Kamori, Mtebe Majigo

**Affiliations:** Department of Microbiology and Immunology, Muhimbili University of Health and Allied Sciences, Dar es Salaam, Tanzania

**Keywords:** *T. gondii*, HIV women

## Abstract

**Background:**

Toxoplasmosis in HIV-infected women of child-bearing age (HIV-WCB) increases the risk for congenital toxoplasmosis, leading to many complications. However, its magnitude is unknown in this population. Objectives: The study aimed to determine the prevalence and factors associated with toxoplasmosis among HIV-WCB.

**Methods:**

This was a cross-sectional study conducted from July to August 2020 among HIV- WCB attending care and treatment clinic (CTC) at Muhimbili National Hospital and Mnazi Mmoja hospital. Questionnaire and TORCH rapid test were used to obtain data and serological testing respectively. Data analysis was done using statistical package for social sciences (SPSS) version 20.

**Results:**

Overall, 29.7% of the study participants were positive for anti-*T. gondii* IgG, whereas none tested positive for IgM. Multivariate analysis showed that the probability of being infected with *T. gondii* increased by 57.1% for participants who consumed raw vegetables (p=0.005, aOR=0.43, 95%CI = 1.24–8.77). Other common risk factors such as undercooked meat consumption, source of drinking water, and cat ownership at home showed no association.

**Conclusion:**

A high number of HIV-WCB have not developed immunity to *T. gondii* in the study area. Introduction of routine screening during antenatal visits for pregnant women and further epidemiological studies are warranted in the country.

## Introduction

Toxoplasmosis is a parasitic infection caused by *Toxoplasma gondii*, which is common among immunocompromised individuals such as people living with HIV (PLHIV)^1^. The parasite infects a wide range of mammals especially cats and other felines. The parasite is transmitted to humans when they ingest excreted oocytes contaminating water, raw vegetables, or undercooked meat^2^. Furthermore, the parasites in infected pregnant woman can cross the placenta to affect the foetus's development, leading to congenital toxoplasmosis resulting to psychomotor disturbances and hydrocephaly or microcephaly^3^,^4^. The congenital toxoplasmosis has many complications such as spontaneous abortion, stillbirth, mental retardation, or neonatal death after delivery^3^,^4^,^5^.

Globally the prevalence of toxoplasmosis ranges from less than 10% in some areas to 95% in others^2^,^6^. In sub-Saharan Africa, the prevalence of toxoplasmosis ranges from 44% to 83%, while the incidence rate of congenital toxoplasmosis is approximately 1.5 cases per 1000 live birth.^6^ In Tanzania, the prevalence of toxoplasmosis among blood donors, is reported to be 47.5%, signifying a public health problem.^7^

Several risk factors including the socioeconomic status and cultural habits of the community, health care education and economic status, geographical factors, cat lifestyle and density, wildlife structure and climate conditions have been documented to be associated with toxoplasmosis.^8^ Despite the complications brought about by congenital toxoplasmosis, routine screening in pregnant women is not done in Tanzania. Lack of routine screening is risky, especially for PLHIV who can develop an active illness from reactivation of prior infection due to compromised immunity^9^. Therefore, we carried out the study to determine the magnitude and factors associated with detection of toxoplasmosis seropositivity among HIV-WCB attending HIV care and treatment clinic (CTC) in Dar es Salaam, Tanzania. The results can be used as a baseline data to carry out large scale studies to decide whether its high time to introduce toxoplasmosis screening as a routine test among HIV-WCB, to help avoid congenital transmission and its associated complications.

## Methods

### Study site and design

This was a hospital-based cross-sectional study conducted from July to August 2020 among HIV- WCB attending CTC at Muhimbili National Hospital (MNH) and Mnazi Mmoja hospital. MNH is the largest referral hospital in Tanzania, and a university teaching hospital with a CTC unit that serves over 800 patients per month. Mnazi Mmoja hospital is a district hospital in Ilala municipal in Dar es Salaam region which has a CTC unit serving about 300 patients per month.

### Study population

The study enrolled 296 HIV-infected women of reproductive age (15 to 49 years) as per world health organisation (WHO) guideline^10^. Participants who granted written consent were enrolled in the study consecutively until the sample size was met. All women within the defined age range attending the health facilities on the day of sample collection had the same chance of being included. Women who were sick and suspected/confirmed of other infectious diseases were excluded in order to avoid affecting the specificity of the test.

### Study variables

The dependent variable was the presence anti-*T. gondii* antibodies. Independent variables were age, education level, eating habits (meat and vegetable consumption preferences) and ownership of cats at home. Consumption of undercooked foodstuffs like meat and raw vegetables was defined as consumption of the same at least once every week in the past one month.

### Data collection

Standardized questionnaire was the tool used to obtain socio-demographic information. The information recorded included; age, education level, and commonly known factors associated with toxoplasmosis, such as eating undercooked meat and raw vegetables, owning cats, and the source of drinking water^11^. Also, whole blood was collected from consented participants by fingertip puncture.

### Laboratory investigations

Onsite TORCH Panel Rapid Test (CTK Biotech, Inc) that tests both anti-*T. gondii* IgG and IgM were used to detect the presence of anti- *T. gondii* antibodies. Two drops of blood were dispensed on the sample well, followed by the addition of 2 drops of sample diluent. Reading of results was done after 10 minutes following manufactures instructions3. Considering its limit of detection, the sensitivity of the test was 2.5 IU/ml, with an accuracy of 94.9% and 98.8% for anti-*T. gondii* IgG and IgM, respectively.

### Data Analysis

All statistical analysis was done using SPSS software V20. In this study seroprevalence of *T. gondii* was obtained by diving the total number of women who tested positive for IgG out of the number of total women enrolled, the result was then presented in percentage. Other descriptive analysis was done to describe parameters including sociodemographic data i.e., age, education whereby, the categorical variables were summarized in form of frequencies, and percentages. Pearson's Chi-square test was performed to observe the significance of proportion differences and p value of less than 0.05 was considered to be statistically significant. The association between socio-demographic characteristics and seropositivity was measured using bivariate and multivariate analysis, whereas the association was regarded significant for a p-value <0.05. All factors with a p value of ≤0.2 and those which have been documented by others to be associated with toxoplasmosis (e.g., level of education, eating habits and ownership of cats) regardless of the p value were subjected to multivariable analysis.

### Ethical consideration

This study was approved by the Institutional Review Board (IRB) of Muhimbili University of Health and Allied Sciences. Permission to conduct the study in the mentioned hospitals was sought from the respective hospital administration. The study participants gave informed written consent to participate after being given detailed information about toxoplasmosis, and the benefits of being part of the study. The whole test procedure was explained to the participants. Participant's data was kept confidential, and special codes and numbers were used instead of names. The test results were shared with participants.

## Results

### Social demographic characteristic of study participants

A total of 296 HIV-infected women aged 15–49 years old were included. The majority of study participants (235/296; 79.4%) were aged 33 years old and above. Most participants (184/296; 62.2%) had a primary education level. More than half (173/296; 58.8%) reported consumption of undercooked meat in some of their meals. About three quarter (229/296; 77.4%) of the participants used tap water, and only few (102/296; 34.5%) had cats at their home. Almost 63.9% (189/296) of the study participants had a habit of consuming raw vegetables (Table 1).

**Table 1:** Socio-demographic characteristic of study population

Variable	Frequency (%)
**Median age = 40**	
**Age group (years)**	
15–20	15 (5.1)
21–26	21 (7.1)
27–32	25 (8.4)
33 and above	235 (79.4)
**Education level**	
Primary education	184 (62.2)
Secondary education	73 (24.7)
Post-secondary education	39 (13.2)
**Consumption of undercooked meat**	
Yes	173 (58.8)
No	121 (41.2)
**Source of drinking water**	
Tap water	229 (77.4)
Water of the well	58 (19.6)
Other	9 (3.0)
**Having cats at home**	
Yes	102 (34.5)
No	194 (65.5)
**Consumption of raw vegetable**	
Yes	189 (63.9)
No	107 (36.1)

### Seroprevalence of toxoplasmosis among study participants

All 296 participants were tested for anti-*T. gondii* IgG and IgM. The seroprevalence toxoplasmosis was 29.7%, of which all positive cases were for IgG, none of the participants tested positive for IgM. In comparison to other age groups, women between the ages of 21–26 had a higher seroprevalence of 38.1%, but the difference was not statistically significant (p=0.27). Participants who had primary education level had a high prevalence (31.5%) of anti-*T. gondii* IgG compared to those who had higher education level, and the difference was not statistically significant (p=0.67). There was almost equal distribution in the detection of anti-*T. gondii* IgG antibodies among participants who used tap water, well, and other drinking water sources (31%,25.9%, and 22.2%, respectively). There was no difference in the prevalence of anti-*T gondii* IgG among participants who consumed undercooked meat (30.6%) and those who did not (28.9%) (p=0.75) (Table 2).

**Table 2:** Distribution of toxoplasmosis among study participants

Variable	Frequency(N)	Serostatus (IgG)		p- value
		Positive n (%)	Negative n (%)	
**Overall**	296	29.7	70.3	
**Mean age 38; SD 8**				
**Age groups (years)**				
15–20	15	2 (13.3)	13 (86.7)	
21–26	21	8 (38.1)	13 (61.9)	0.27
27–32	25	5 (20)	20 (80)	
33 and above	235	73 (31.1)	162 (68.9)	
**Education level**				
Primary education	184	58 (31.5)	126 (68.5)	0.67
Secondary education	73	19 (26)	54 (74)	
Post-secondary education	39	11 (28.2)	28 (71.8)	
**Source of drinking** **water**				
Tap water	229	71 (31)	158 (69)	0.66
Well water	58	15 (25.9)	43 (74.1)	
Other	9	2 (22.2)	7 (77.8)	
**Consumption of** **undercooked meat**				
Yes	173	53 (30.6)	120 (69.4)	0.75
No	121	35 (28.9)	86 (71.1)	
**Consumption of raw** **vegetable**				
Yes	189	67 (35.4)	122 (64.6)	0.004
No	107	21 (19.6)	86 (80.4)	
**Having cats at home**				
Yes	102	27 (26.5)	75 (73.5)	0.37
No	194	61 (31.4)	133 (68.6)	

### Factors associated with toxoplasmosis

Women who had habits of consuming raw vegetables were two times more likely to have anti-*T. gondii* IgG compared to those who did not consume raw vegetables (p = 0.005, aOR = 0.429, 95%CI = 0.239–0.772). There was no association between having a cat at home, consumption of raw meat, source of water and detection of anti-*T. gondii* IgG (Table 3).

**Table 3:** Logistic regression analysis of factors associated with anti-*T. gondii* positivity

Variable	Anti *T.* *gondii* antibody Positive n (%)	Univariable			Multivariable		
		cOR	95% CI	p- value	aOR	95%CI	p-value
**Age**							
15–20	2 (13.3)	1					
21–26	8 (38.1)	0.25	0.04–1.41	0.12	0.27	0.44–1.61	0.15
27–32	5 (20.0)	0.62	0.10–3.66	0.59	0.83	0.13–5.19	0.84
33 and above	73(31.1)	0.34	0.08–1.55	0.16	0.40	0.08–1.96	0.26
**Education** **level**							
Primary education	58(31.5)	0.85	0.40–1.83	0.68	0.96	0.43–2.14	0.91
Secondary education	19(26)	1.12	0.47–2.67	0.80	1.08	0.43–2.71	0.87
Post-secondary education	11(28.2)	1					
**Source of** **drinking** **water**							
Tap water	71(31)	1					
Well water	15(25.9)	1.29	0.67–2.47	0.45	1.39	0.70–2.72	0.35
Other	2(22.2)	1.57	0.32–7.76	0.58	2.06	0.37–11.39	0.41
**Consumption** **of** **undercooked** **meat**							
Yes	53(30.6)	0.92	0.55–1.53	0.75	0.98	0.58–1.66	0.94
No	35(28.9)	1			1		
**Consumption** **of raw** **vegetable**							
Yes	67(35.4)	0.45	1.25–8.78	0.005	0.43	1.24-8.77	0.005
No	21(19.6)	1			1		
**Having cats** **at home**							
yes	27(26.5)	1.27	0.75–2.17	0.37	1.05	0.60–1.84	0.87
No	61(31.4)	1			1		

cOR = crude odds ratio, aOR = adjusted odds ratio, CI= Confidence interval, 1= reference category

## Discussion

The current study was designed to determine the seroprevalence of toxoplasmosis among HIV-infected women of child-bearing age attending HIV care and treatment clinics in Dar es Salaam. The overall seroprevalence of anti-toxoplasmosis IgG was 29.7%, and none of the participants tested positive for IgM. A similar trend has been previously reported among Sudanese women which was 27%^12^. However, the reported prevalence is much lower compared to a review report from Ethiopia, which reported a seroprevalence of 85.7% among HIV-infected people and 72.5% among pregnant women. The high seroprevalence reported in Ethiopia might partly be due to early childhood and teenage infection in that region and the use of a more sensitive detection methods (ELISA and latex agglutination test (LAT)) in some studies which could detect all cases contrary to our testing method, which is less sensitive compared to those methods. 8

In this study, higher prevalence of toxoplasmosis was observed among the middle-aged group of 21–26 years old and those with a primary education level, although the difference was not statistically significant. Similar results have been reported among pregnant women in Northern Iran^13^. It is widely accepted that a higher educational level usually means more knowledge about the infection and its prevention hence those with lower education have a higher chance of getting transmissible infections.

In line with studies from Tanzania and Australia, consuming raw vegetables was significantly associated with the detection of anti-*T. gondii* antibodies, with a 57% more chance of detecting anti-*T. gondii* antibodies among those who consumed raw vegetables^14^,^15^. The reason could be that most of the vegetable is contaminated with oocysts from the soil in the gardens.

Based on the source of water, our study observed a high seroprevalence of anti-*T gondii* among participants using tap water for drinking. However, this association was not statistically significant, just as reported in other studies^14^,^16^. The possible reason for this is that the study was done in an urban area where there is a good supply of water services by the urban authority, unlike other studies that involved a mixed population from both urban and rural areas.

Other risk factors for toxoplasmosis, such as eating undercooked meat, showed no significant association to anti-*T. gondii* prevalence contrary to what has been reported in many other studies^17^,^8^. The reason for this difference could be due to participants' subjectivity on whether the consumed meat was undercooked.

The small number of study participants enrolled in this study is among the limitations of this study. Due to the small sample size, the results might not correctly present the magnitude of toxoplasmosis among the study population. Also, responses to some questions were subjective to the participant's perception since there was no set cutoff values for some factors.

## Conclusion

There is a low number of HIV-infected women of child-bearing age in Dar es Salaam who have not developed immunity to toxoplasmosis putting them at risk of getting acute infection. Therefore, in line with routine screening to detect and manage toxoplasmosis cases, education should be provided in the clinics to prevent them from getting infected. However, further studies should be done to explore more on toxoplasmosis and its risk factors to obtain more information that can be used to improve and promote the public's good health.

## Abbreviations

CTC: Care and treatment clinic
ELISA: Enzyme-linked immunosorbent assay
HIV: Human immunodeficiency virus
HIV-WCB: HIV infected women of child bearing age
IgG: Immunoglobulin G
IgM: Immunoglobulin M
KCMC: Kilimanjaro Christian medical Centre
MNH: Muhimbili national hospital
SPSS: Statistical Package for the Social Sciences
TORCH: Toxoplasma, rubella, cytomegalovirus, and herpes simplex virus
